# Musical Dogs: A Review of the Influence of Auditory Enrichment on Canine Health and Behavior

**DOI:** 10.3390/ani10010127

**Published:** 2020-01-13

**Authors:** Abigail M. Lindig, Paul D. McGreevy, Angela J. Crean

**Affiliations:** 1 Sydney School of Veterinary Science, The University of Sydney, Sydney, NSW 2006, Australia; alin9343@uni.sydney.edu.au (A.M.L.); paul.mcgreevy@sydney.edu.au (P.D.M.); 2 School of Life and Environmental Sciences, Charles Perkins Centre, The University of Sydney, Sydney, NSW 2006, Australia

**Keywords:** dog, shelter, enrichment, classical music, stress, music therapy, behaviour

## Abstract

**Simple Summary:**

Interest in the use of music therapy as a behavioral enrichment tool in veterinary medicine is growing. Indeed, an industry has formed around the development of ‘dog music’, which has been purposely designed to relax dogs. Despite enthusiastic uptake of the idea, there is little empirical evidence supporting the design of such tools. This article summarizes the scientific literature in this emerging domain. It notes that, as a general observation, animals appear less stressed or anxious when exposed to classical music than to control conditions. It also acknowledges that this field is relatively under-researched, and more rigorous studies must be conducted before species-specific recommendations can be made. Such studies must reflect individuals’ and species’ preferences for different genres and songs, taking care to avoid habituation.

**Abstract:**

Music therapy yields many positive health outcomes in humans, but the effects of music on the health and welfare of nonhuman animals vary greatly with the type of music played, the ethology of the species, and the personality and learning history of individual animals. One context in which music therapy may be used to enhance animal welfare is to alleviate stress in domestic environments. Here, we review studies of the effects of music exposure on dogs as a case study for the implementation of music therapy in veterinary medicine. Nine reports of experimental testing for the therapeutic effects of music on dogs were found, with most of these studies focusing on changes in behavior. Overall, exposure to classical music appears to have a calming influence on dogs in stressful environments, with no additional benefit observed from any music purposely designed for dogs (specifically “Through a dog’s ear”). Given the cost effectiveness and ease of implementation, music therapy holds promise in veterinary medicine and animal welfare. However, to address precise research questions, further studies must use clearly defined characteristics of stimulus music in the experimental design, and consider the variability of each individual animal’s physical characteristics and past experience in the selection of candidates.

## 1. Introduction

Music as a therapy for humans has been shown to yield many positive outcomes, such as pain relief, reduced blood pressure, heart rate and anxiety levels [1,2,3]. Due to the low cost and the ease of administration of music to domestic animals, there is growing interest in adapting music therapy techniques from the human domain to improve the health and welfare of companion, performance and production animals [4]. However, when assessing the effect of music upon nonhuman animals, it is important to consider how each species has evolved to encounter auditory stimuli, the type of music administered, the desired outcome, and the individual differences among animals. Studies of comparative acoustics have shown that the anatomy of the head, the distance between the ears and the shape and mobility of the pinnae influence the effective acoustic radius [5]. Even among a single species, such as *Canis lupus familiaris*, individuals’ size, head shape, the shape and mobility of the pinnae [6] and learning history [7] can create variation that would affect perception and outcomes. Hence, music therapy cannot be simply translated from the human context into veterinary medicine; carefully designed experiments are required to implement evidence-based practice.

There are several potential benefits of implementing music therapy for domestic animals. For example, music therapy may have a positive effect upon the health and behavior of an individual or group of animals in a stressful context (e.g., veterinary hospital, abattoir, milking parlor, boarding kennel, zoological park) (reviewed in [4]). Music may also be used to increase production in agriculture systems. For example, it has been effectively used to encourage dairy cows to approach the stalls in an automatic milking parlor [8]. Furthermore, cows exposed to classical music in the milking parlor showed fewer behavioral indicators of stress, and produced more milk than cows exposed to only the noises of the automated system. So, for the period of this study at least, music used in this context reaped a dual benefit of increased profit and animal welfare [8]. Importantly, auditory enrichment is relatively cheap and easy to employ. In contrast to many other forms of environmental enrichment that can often require additional materials, food, and labor to construct, music can be easily administered via a speaker system. 

Important considerations in the implementation of music therapy are the characteristics of the species and individuals being targeted. The effect of music on animal health and welfare may also depend on the music’s genre, tempo, or instrumentation of the music played. While many animals have been shown to benefit from music exposure, others have shown adverse reactions or no changes in behavior when exposed to music. For example, behavioral observations in captive cotton-topped tamarins (*Saguinus oedipus*, *n* = 5) and common marmosets (*Callithrix jacchus*, *n* = 4) indicated that they preferred silence to both fast and slow music [9]. In a study of captive Moloch gibbons (*Hylobates moloch*, *n* = 8), the use of auditory enrichment in an animal park induced no significant differences in activity, brachiation and affiliative behaviors, such as grooming [10]. In another example, ‘fast music’ (rock music) was shown to decrease daily growth rates in pigs, while ‘slow music’ (light music) had no effect upon growth rate [11]. Finally, animals may become habituated to music, and therefore any effects may decrease over time [12]. Hence, properly controlled experiments, designed with the target population and outcome response clearly defined, are required to achieve the best outcome when implementing music therapy in veterinary medicine.

The use of music therapy in nonhuman animals has been examined most thoroughly in companion animal species, especially dogs. Hence, here we systematically review experimental studies of music exposure in dogs as a case study to inform future directions for the implementation of music therapy in more general attempts to boost animal health and welfare. The current data suggest that using music as an instrument to improve welfare requires a multifactorial approach, with consideration of species, individual differences, genre and composition of music, background noise, and long-term experimental data collection to guide these choices.

## 2. Materials and Methods

**Figure 1:**
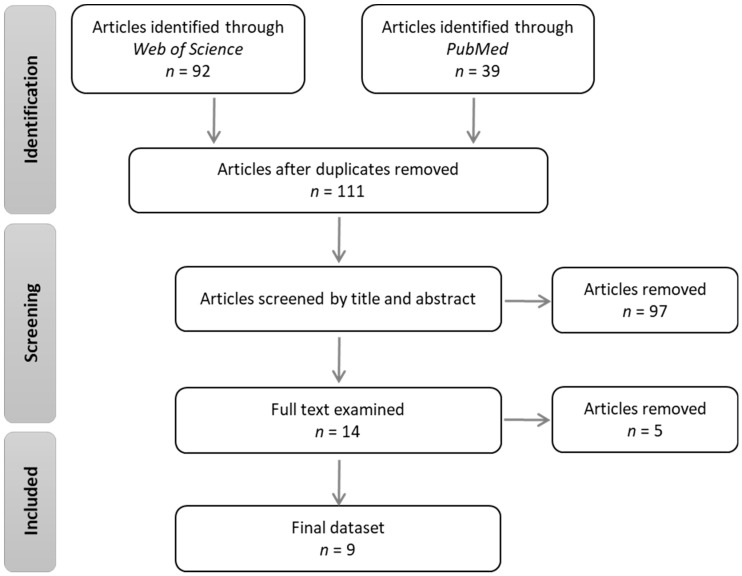
Preferred Reporting Items for Systematic Reviews and Meta-Analyses (PRISMA) flow diagram of study selection.

A literature search with the strings “music AND dog OR canine” was completed on 31 October 2019 in the ISI Web of Science and PubMed databases. These databases were selected because of their high volumes of peer-reviewed scientific literature on animal health, science and behavior. This search yielded 111 unique articles, whose titles and abstracts were screened for relevance by two independent reviewers (see Figure 1). Only studies that experimentally manipulated the auditory environment and assessed at least one measure of canine health or behavior were included for further review, leaving 14 articles for full-text screening. 

Five of these articles (all by the same team of authors and published between 1987–1989) were then excluded, because music was used to induce stress rather than enrichment. This process left nine articles in the final dataset (Table 1). 

Follow-up searches of music therapy studies in other species were conducted by combining general and more specific key words relating to music therapy, auditory enrichment, and its behavioral and physiological effects on animals. Search terms included combinations of ‘auditory’, ‘enrichment’, ‘stress’, ‘animal’, ‘music’, ‘immun*’, ‘husbandry’, ‘health’, ‘behav*’ and ‘welfare’. Only publications in English were included. A sample of the range of studies examining the effects of music on animals is shown in Table 2. This sample is not comprehensive, nor part of the systematic review. Rather, we highlight these studies to provide new ideas for future research in the effects of music on canine health and welfare. 

## 3. Results

Nine studies experimentally testing the therapeutic effects of music on dogs were examined (Table 1). Most of these studies are recent (7/9 studies having been published in the past five years, with the remaining two having been published in 2002 and 2012), demonstrating an escalating interest in this emerging area of research. The studies cover a range of breeds, across all ages from puppies to adults, and generally examine both sexes. Settings include animal shelters, veterinary hospitals and police-dog training facilities. All but one study examined behavioral measures as a response variable, with most including an assessment of time spent resting and barking (among other variables) as indicators of stress. Six of these eight studies reported that exposure to music affected one or more behavioral measures, with most revealing that classical music had a calming effect compared to controls and other forms of music (Table 1). Fewer studies examined health and/or physiological measures. Three studies examined heart rate variability, with all three finding significant changes with exposure to music [12,17,20]. Two studies examined circulating cortisol concentrations, with one finding no difference in salivary cortisol concentrations as a result of exposure to classical music [12], and the other finding increased urinary cortisol concentrations with exposure to soft rock music [17].

## 4. Discussion

There is growing interest in using music therapy as a behavioral management and enrichment tool in veterinary medicine [4]. In particular, an industry has formed around the development of ‘dog music’, which has been purposely designed to relax dogs. Despite an enthusiastic uptake of the idea, there is little empirical evidence supporting the design of such tools. A broad search of the terms ‘music’ and ‘dog’ only yielded nine studies that experimentally tested the effects of music exposure upon dogs (Table 1). Overall, these studies provide strong evidence that exposure to music alters the behavioral traits of dogs, with classical music generally being reported to have a calming influence in potentially stressful environments such as boarding kennels, rescue shelters and veterinary clinics. Overall, classical music was associated with dogs spending more time sitting or lying down, resting and sleeping, and less time vocalizing and standing (Table 1). However, music marketed as being specifically designed for dogs (the five studies examining dog music all used “Through a dog’s ear”) did not appear to have many beneficial effects over and above those gained by exposing dogs to a random selection of classical music [14,15,16,18,20]. This is likely due to the heterogeneity of the populations studied, and/or a lack of sensitivity in the response variables measured. To develop evidence-based guidelines for the implementation of music therapy in veterinary medicine, further experiments using carefully controlled target populations, interventions and response measures are required.

Of the nine studies identified in our systematic review, all but one study assessed behavior as the main response variable. Only four of the nine studies tested for the physiological effects of music exposure in dogs, providing some supporting evidence of the interpretation from behavioral data of a reduction in stress levels during exposure to classical music [12,17]. Yet, the beneficial effects of music therapy in canine shelters is likely to extend far beyond the limited responses reported to date. Alternative study designs and response measures examined in other animal species may be adapted to inform future directions (Table 2). For example, additional benefits of music therapy in animal shelters may include beneficial effects on immune function and metabolism [21,25]. Exposure to classical music has been shown to enhance immune function and anti-tumor responses in laboratory rodents (both mice and rats) [21], and immunity and developmental stability in layer chicks [23]. Investigations into the effects of the exposure of veterinary patients to music in clinics have begun [16,18,20], but such studies have thus far been limited by small sample sizes. Another potential use of music in animal shelters is to influence animal movement patterns [8,24] by drawing towards certain areas and repelling them from others. Theoretically, this has the potential to facilitate the provision of larger, relatively free-range enclosures. In addition, music in animal shelters and veterinary hospitals may have flow-on benefits to staff and visitors [18], and may improve adoption rates [13,17]. Hence, in addition to improving the immediate and short-term physiological and mental health of dogs, music therapy may improve long-term welfare, potentially countering the effects of overcrowding and protracted stays in shelters. We encourage future studies to broaden the scope of response variables examined to gain insight into the full range of health and welfare benefits gained through music therapy.

While the addition of classical music to kennel and veterinary hospital environments seems to yield positive benefits overall, several considerations arise when using auditory enrichment as a welfare measure. Four of the studies compared the effects of various broad categories of music including Heavy Metal [13,14], Pop [13,15,17] and Soft Rock, Motown and Reggae [17]. Rock and heavy metal music were found to induce undesirable behavioral and physiological changes in dogs, such as increased barking/vocalizing and standing [13,14,17]. However, it is still not clear what specific traits of each type of music stimulus are responsible for the altered outcomes. Some generalizations can be inferred, and traits such as pitch, tempo and volume have been considered. Yet, the category of music classified under the umbrella term ‘classical’ is wide and varied. Future studies should consider traits such as instrumentation, as the timbre of the music may affect outcomes. For example, a study that examined piglets’ reactions to different types of classical music revealed differences between the effects of fast and slow tempos, as well as between string and wind instruments [24]. Slow string and wind music were both associated with more exploratory behaviors, although slow string music also resulted in more time spent lying down [24]. Fast wind music significantly increased the time piglets spent walking, lying, standing and exploring [24]. Responses to music instrumentation is likely to vary among species, and therefore further research is required to determine if similar affects are found in dogs. In addition to instrumentation, a final consideration of variation within the category of classical music is the inclusion of a human voice. Praising words combined with a praising intonation activate primary reward regions in dog brains [26]. Furthermore, Brayley and Montrose [15] found that playing an audiobook induced calmer behavior than both classical and dog music. Hence, it would be interesting to investigate the effects of instrumental versus vocal classical music, particularly when the text includes praising words.

Another important consideration is that of the physical differences among breeds, as head shape and anatomy can alter the perception and responses to music treatments [6]. Similarly, previous experiences of each individual animal may have conditioned positive or negative associations with a particular type of music that will influence the response of that particular animal [7]. The nine studies examined covered a range of dog breeds and ages, and generally included both males and females, so it is interesting that over this wide range of animals, general patterns of changes in behavior were observed. However, Bowman et al. (2015) reported that heart rate variability measurements were not consistent throughout the trial, suggesting that dogs may have individual preferences for certain songs [12]. It is important to recognize that humans also have auditory physiological and anatomical differences from other species, and so music that is generally calming to humans may not have the same influence on nonhuman animals [4,27,28,29]. Moreover, some individuals may prefer silence to music. For example, stress behaviors (e.g., pacing) and plasma cortisol concentrations were found to decrease in captive primates that were granted the facility to choose when the music was on or off [4]. Similarly, in humans, autonomy and personal preference are crucial factors in music’s ability to alleviate stress [25]. Therefore, it is important to give individual animals autonomy over their musical environment (e.g., by providing music in one part of the kennel and relative quiet in another area). In addition, consideration should be given to variability in the playlist used. Bowman et al. (2015) found that dogs exposed to the same playlist repeatedly over 7 days became refractory to the psychological and physiological effects of the music [12]. This rapid habituation can be moderated by increasing variety and changing the sequence in which the tracks are presented [17]. 

## 5. Conclusions

The use of music in domestic animal contexts shows great potential as an effective and easy-to-implement therapeutic measure with many flow-on benefits to animal health and welfare. When considering auditory enrichment for the improved welfare of animals in stressful environments, it is important to consider a few key factors. Firstly, this field is relatively under-researched, and more empirical studies must be conducted before specific recommendations can be made. The elimination of ultrasound and infrasound that may distress nonhuman listeners is a basic first step. As a general observation, animals appear less stressed or anxious when exposed to classical music than to control conditions e.g., [8,17,23,24]. However, it is important to consider and adapt to individuals’ and species’ preferences for different genres and songs, taking care to avoid habituation [17]. Overall, the addition of classical music as an enrichment of environments in which animals are confined or potentially under stress is a relatively inexpensive and easily implemented enrichment that appears to withstand cost-benefit analysis. It offers potentially considerable benefits in terms of animal behavior, health and welfare [8,14]. Owners, trainers, keepers and managers should therefore consider and assess the potential benefits of using auditory enrichment for animals in their care.

## Figures and Tables

**Table 1:** Studies examining effects of auditory enrichment on canine health and behavior.

Authors, Year, Ref	Population	Location	Intervention	Measures	Result
Wells, Graham, Hepper 2002 [13]	*n* = 50 male: 27 neutered female: 23 spayed Age from 6 m–6 y mainly cross-bred	Rehoming Center, UK	Five treatments: 1. Control; 2. Human conversation (radio program); 3. Classical music (mix of tracks); 4. Heavy metal music (mix of ‘Metallica’ tracks); 5. Pop music (mix of tracks). Dogs exposed to each of the five interventions on five separate days, with one day rest in between. Acoustic stimuli presented between 10:00–14:00.	Three behavioral parameters observed: Position in kennel, activity, and vocalization. Dogs observed at 10 min intervals for duration of intervention.	More time spent at front of kennels during all auditory interventions. Changes in activity and vocalization related to type of auditory stimulation—more time resting and quiet when exposed to classical music. Increased barking during heavy metal music exposure.
Kogan, Schoenfeld-Tacher, Simon 2012 [14]	*n* = 117 Two groups: 1. rescue dogs male: 21 neutered female: 12 spayed, 1 entire Mean age 5.27 y Dachshund (pure and cross) 2. short-term boarding male: 31 neutered, 10 entire female: 38 neutered, 4 entire Mean age 5.92 y Variety of pure and mixed breed	Dog shelter and boarding facility, USA	Four treatments: 1. Control; 2. Classical music (4 tracks); 3. Heavy metal music (3 tracks); 4. Dog relaxation music (Through a dog’s ear (TADE)). 45 min exposure, followed by a 15 min break. Three conditions tested per day between 09:00–12:00 Dogs exposed over 4 months, not all dogs exposed to all treatments.	Three behavioral parameters observed: Activity, vocalization, and body shaking. Dogs observed at 5 min intervals for duration of intervention.	No interaction between treatment and group (rescue vs. boarding dogs), although rescue dogs spent more time sleeping and silent. Changes in activity and vocalization related to type of auditory stimulation—more time sleeping and quiet when exposed to classical music. Increased body shaking during heavy metal music exposure.
Bowman, Dowell, Evans, Scottish 2015 [12]	*n* = 50 male: 25 neutered, 9 entire, female: 12 spayed, 4 entire Age from <1 y to >10 y Variety of breeds, high proportion of Staffordshire bull terriers Aggressive dogs excluded	Animal rescue and rehoming center, Scotland	Two treatments: 1. Control; 2. Classical music (mixed tracks, low pitch and slow tempo) Dogs exposed to one treatment for 7 days, then the alternate treatment for 7 days. Acoustic stimuli presented between 10:00–16:30.	Three behavioral parameters: Position, location, and vocalization. Heart rate variability. Salivary cortisol. Observations made for 1.5 h, 2x/day (10:30–12:00 and 14:00–15:30), on days 1, 7, 8 and 14. Saliva collected at the end of each 1.5 h period.	Classical music exposure induced changes in behavior (less time standing and barking) and altered heart rate variability, indicative of reduced stress. No significant changes in salivary cortisol. Resistance to effects of music if the same playlist is used repeatedly. Therapeutic effects of music more obvious in male dogs.
Brayley, Montrose 2016 [15]	*n* = 31 male: 24 neutered female: 7 spayed Age from 9 m to 13 y	Rescue shelter, UK	Five treatments: 1. Control; 2. Audiobook; 3. Classical music (Beethoven); 4. Pop music (mix of tracks); 5. Dog relaxation music (TADE). Dogs exposed to each treatment for 2 h, with 2 days rest in between. Acoustic stimuli presented between 10:00–12:00.	15 behaviors sampled, including multiple measures of activity and vocalization. Behavior recorded every 5 min using an ethogram.	Audiobook treatment induced calmer behavior than all other treatments. Classical music induced calmer behavior than pop music and no intervention (control).
Albright, Seddighi, Ng, Sun, Rezac 2017 [16]	*n* = 10 male: 8 neutered, 2 entire Mean age 4.2 y Beagles	Veterinary hospital, USA	Five treatments: 1. negative control (saline); 2. positive control (sedative); 3. Human voice quiet (55–60 dB); 4. Human voice loud (80–85 dB); 5. Dog relaxation music (TADE) (45–50 dB). Dogs exposed to treatment for 20 min after sedative injection. Each dog exposed to all treatments with a minimum of 48 h rest in between. Testing between 08:00–15:00	Depth of sedation assessed by spontaneous behavior, accelerometry, and restraint tests. Behavior assessed every 5 min and compared before, during and after treatment. Restraint tests 30 min and 40 min post-treatment	Sedation is negatively impacted by high-intensity noise conditions (80–85 dB) Exposure to music marketed as having a calming effect in dogs did not improve sedation
Bowman, Dowell, Evans, Scottish 2017 [17]	*n* = 38 male: 15 neutered, 9 entire, female: 7 spayed, 7 entire Age from <1 y to >8 y Variety of breeds, high proportion of Staffordshire bull terriers	Animal rescue and rehoming center, Scotland	Six treatments: 1. Control (before and after); 2. Soft rock; 3. Motown; 4. Pop music; 5. Reggae music; 6. Classical music Dogs exposed to a different music treatment each day for 5 consecutive days. Acoustic stimuli presented between 10:00–16:00.	Three behavioral parameters: Position, location, and vocalization. Heart rate variability. Urinary cortisol. Observations made for 1 h, 2x/day (10:30–11:30 and 14:30–15:30). Urine collected between 13:00–14:00 on days 1, 4–8 and 11.	Music exposure (all genres) induced changes in behavior (less time standing) and altered heart rate variability, indicative of reduced stress. Urinary cortisol higher during soft rock exposure. Physiological and behavioral changes were maintained over the 5 d of auditory stimulation, suggesting that providing a variety of different genres may minimize habituation.
Engler, Bain 2017 [18]	*n* = 74 no further information supplied.	Veterinary teaching hospital, USA	Three treatments: 1. Control; 2. Classical music; 3. Dog relaxation music (TADE) Treatment rooms assigned to each treatment. Music played continuously throughout the day. Each dog only exposed to a single treatment (control = 30, music = 23, TADE = 21)	Owners and clinicians completed a standardized survey regarding dogs’ behavior. Physiological variables obtained from the medical record.	No difference in behavior (aggression, anxiety) or physiology (body temperature and heart rate) of dogs detected. Classical music had a positive effect upon owner and employee satisfaction.
Alves, Santos, Lopes, Jorge 2018 [19]	*n* = 67 males: 34 females: 33 Puppies (7 weeks old) Four different breeds (27 German shepherd dogs, 19 Belgian Malinois shepherd dogs, 7 Dutch shepherd dogs, and 14 German shepherd dog and Belgian Malinois shepherd dog crosses).	Police canine unit, Portugal	Two treatments: 1. Control (*n* = 46); 2. Varied auditory stimulation (including music, radio talk shows, and environmental noise (cars, sirens, gunshots)) (*n* = 21) Auditory stimulation provided from 3 weeks of age for 2 h/day, focused around meal and play time.	Puppies’ performance in a skills test (9 scenarios ranging from following and submission to startle response and pain sensibility) evaluated at 7 weeks of age.	Auditory stimulation had a negative effect upon puppies’ performance overall, in particular on following, lifting by evaluator, and submission tests.
Koster, Sithole, Gilbert, Artemiou 2019 [20]	*n* = 16 (8 kenneled, 8 student-owned) male: 5 female: 11 Mean age 3 y 81% mixed breed	Veterinary teaching hospital, West Indies	Two treatments: 1. Control; 2. Dog relaxation music (TADE) All dogs exposed to one treatment for a 60 min veterinary training session, then 7 days later exposed to the alternate treatment.	Heart rate variability	Auditory stimulation reduced RR variability, suggesting the novel music exposure had an excitatory rather than a calming effect.

**Table 2:** Sample of studies examining effects of auditory enrichment on health and behavior of non-canine species.

Ref	Species	Location	Intervention	Measures	Result
Uetake, Hurnik, Johnson 1997 [8]	Holstein cows (*Bos taurus*) *n* = 19 (mid and late lactating)	Dairy Research Center, Canada	Cows conditioned to identify the start of the milking period when they heard music (Country music) During experimental period cows observed on days with and without music stimulus.	Number of cows in holding area Behavior (standing, eating, resting)	Music stimulates the voluntary approach of cows to holding area and encourages behavioral readiness of cows to milking
Nunez et al. 2002 [21]	BALB/c mice (*Mus musculus*), and Sprague–Dawley rats (*Rattus norvegicus domestica*) *n* = 80 mice (male 7–12 weeks old)	Lab animal facility, Spain	Four treatments: 1. Control; 2. Music; 3. Auditory stressor (fire alarm bell); 4. Auditory stressor and music.	Mice: Immune function (e.g., thymus and spleen cell counts and viability, T cell proliferation) Rats: Injected with carcinosarcoma cells and sacrificed 8 days later to count the number of metastatic nodules on the lungs.	Music enhanced immune parameters and anti-tumor response in unstressed rodents.
Lemmer 2008 [22]	Wistar-Kyoto rats (*Rattus norvegicus domestica*) *n* = 20 (male, 10 normotensive and 10 hypertensive)	Lab animal facility, Germany	Four treatments: 1. Control; 2. White-noise control; 3. Stress response control (cage-switch); 4. Classical music (Mozart/Ligeti)	Radiotelemetry to monitor blood pressure, heart rate, and motor activity. Plasma norepinephrine concentrations	Mozart and Ligeti had different effects on physiology, with more effects detected in hypertensive rats. White noise induced no effects.
Davilla et al. 2011 [23]	Layer chicks (*Gallus gallus domesticus*) *n* = 192 (8 breeds)	Conservation program experimental station, Spain	Two treatments: 1. Control; 2. Classical music (Mozart’s string quartets)	Fear (tonic immobility duration) Stress (heterophil to lymphocyte ratio and fluctuating asymmetry)	Significantly higher heterophil to lymphocyte ratios in chicks reared without music. Significant differences in physical trait measurements (wing length asymmetry, leg width, and combined asymmetry) in birds reared without music. Inconsistent, insignificant effects of music on duration of tonic immobility.
Wallace et al. 2013 [10]	Moloch gibbons (*Hylobates moloch*) *n* = 8 (2 family groups)	Wild Animal Park, UK	Two treatments: 1. Control; 2. Instrumental classical music between 50–90 bpm	Activity Brachiation Affiliative behavior (grooming) Stress behavior (self-scratching)	No significant differences in behavior between music and control treatments.
Li et al. 2019 [24]	Piglets (*Sus scrofa domesticus*) *n* = 35 (males from 14 litters)	Commercial sow farm, China	Five treatments: 1. Control; 2. String instruments, slow tempo (65 bpm); 3. String instruments, fast tempo (200 bpm); 4. Wind instruments, slow tempo (65 bpm); 5. Wind instruments, fast tempo (200 bpm) Piglets given choice between rooms with and without music.	Entries to room Behavior (lying, standing, walking, exploring, feeding, playing, tail wagging)	Piglets spent significantly more time in the slow/string and fast/wind rooms compared to the other music conditions. Behavioral responses varied with music treatments—stronger response to tempo than instrumentation.
